# Community-Acquired Rotavirus Gastroenteritis Compared with Adenovirus and Norovirus Gastroenteritis in Italian Children: A Pedianet Study

**DOI:** 10.1155/2016/5236243

**Published:** 2016-01-14

**Authors:** D. Donà, E. Mozzo, A. Scamarcia, G. Picelli, M. Villa, L. Cantarutti, C. Giaquinto

**Affiliations:** ^1^Division of Paediatric Infectious Diseases, Department of Woman and Child Health, University of Padua, Padua, Italy; ^2^Pedianet Project, Padua, Italy; ^3^Epidemiology Service, Local Health Authority of Cremona, Cremona, Italy

## Abstract

*Background*. Rotavirus (RV) is the commonest pathogen in the hospital and primary care settings, followed by Adenovirus (AV) and Norovirus (NV). Only few studies that assess the burden of RV gastroenteritis at the community level have been carried out.* Objectives*. To estimate incidence, disease characteristics, seasonal distribution, and working days lost by parents of RV, AV, and NV gastroenteritis leading to a family pediatrician (FP) visit among children < 5 years.* Methods*. 12-month, observational, prospective, FP-based study has been carried out using Pedianet database.* Results*. RVGE incidence was 1.04 per 100 person-years with the highest incidence in the first 2 years of life. Incidences of AVGEs (1.74) and NVGEs (1.51) were slightly higher with similar characteristics regarding age distribution and symptoms. Risk of hospitalisation, access to emergency room (ER), and workdays lost from parents were not significantly different in RVGEs compared to the other viral infections.* Conclusions*. Features of RVGE in terms of hospitalisation length and indirect cost are lower than those reported in previous studies. Results of the present study reflect the large variability of data present in the literature. This observation underlines the utility of primary care networks for AGE surveillance and further studies on community-acquired gastroenteritis in children.

## 1. Introduction

Worldwide, acute gastroenteritis (AGE) is the third most common cause of death in children < 5 years of age [1]. In European children, only few deaths occur as a consequence of AGE; however, diarrhoea has a considerable impact on the quality of life of children and their families [2]. Rotavirus (RV) is the leading cause of severe dehydration in children < 5 years of age [3–5].

In the European Union (EU), it is estimated that 3.6 million episodes of RV gastroenteritis (RVGE) occur annually. RVGE is estimated to occur at a rate of 1 symptomatic infection in every 7 children each year, accounting for 231 deaths, more than 87000 hospitalisations, and almost 700000 outpatient visits. It has been estimated that RV accounts for 39% diarrheal hospitalisations [6] and from 25.3% to 63.5% of community-acquired AGE in children < 5 years of age [7–11]. These rates vary greatly depending on whether patients are seen in the hospital, emergency room, or primary care physician clinic.

Most RV infections are community-acquired and transmitted by the fecal-oral route [12] and peak in the winter season between November and February in temperate climates [4, 13, 14].

RVGE imposes a heavy economic burden by incurring not only direct (consultation, emergency, hospitalisation, and medication) costs, but also indirect costs (parent workdays lost, childcare, etc.) [12, 15, 16]. In Europe, it has been associated with direct medical costs per patient ranging from $1942 to $2389. Indirect costs including workdays lost by parents of children hospitalised for RVGE as well as out-of-pocket expenses ranged between $260 and $1061 (UK). A portion of indirect costs was attributed to workdays lost by parents per hospitalisation episode, which varied between 2.3 days and 6.4 days [17].

Since the two RV vaccines became available, a monovalent RV vaccine (Rotarix, GlaxoSmithKline, Rixensart, Belgium) and a pentavalent RV vaccine (RV5, RotaTeq, Merck and Co., Whitehouse Station, NJ, USA; Sanofi Pasteur MSD, Lyon, France), the frequency of RVGE decreased [18], while other pathogens are now reported more frequently. The Italian Society of Pediatrics [19] supports the RV vaccination but this has not been included yet in the national immunization program. However, because the Italian National Health Service is decentralized, regions can include vaccines not nationally recommended in their immunization program.

Moreover, from 2008 into Veneto Region, setting of this study, all mandatory childhood vaccinations have been suspended. Until now, no local data on rotavirus vaccination coverage are available and it is estimated to be very low since rotavirus vaccination is not free of charge like other vaccinations.

The majority of non-RVGEs are usually AGEs associated with Adenovirus (AV) and Norovirus (NV) [20–25]. Also AV gastroenteritis (AVGE) and NV gastroenteritis (NVGE) are predominantly presenting during winter months [26, 27].

Adenovirus (AV), initially recognized as a cause of respiratory disease, is associated also with gastrointestinal, ophthalmological, and neurological infections [28]. The prevalence of AVGE is variable, ranging from 16% to 3.5% [22, 29, 30]. Watery, nonbloody diarrhoea typically precedes vomiting and children admitted to the hospital for AVGE are more likely to present diarrhoea that usually lasts more than in RVGE (more than 5 days) [31, 32].

NV represents the most common cause of gastroenteritis outbreaks and causes acute, self-limiting gastroenteritis in people from all age groups [33].

Three US country surveillances during 2009-2010 showed that 17% of faecal specimens from children (<5 years) hospitalised with gastroenteritis, 23% from children seen in emergency departments, and 28% from children seen in other outpatient settings were positive for NV [34]. Vomiting tends to be more prominent symptom in NVGE gastroenteritis than in other types of viral AGE. Usually, NVGE has milder symptoms than those of RVGE but a higher attack rate, due to its unusual stability outside the host and the low dose needed to produce symptomatic infection [35].

There are limited data on the incidence of AGE pathogens in the primary care setting. In particular, there is an absence of long-term data, per age group on the proportion on RVGE, AVGE, and NVGE among AGE in this setting. This is due to the fact that no systematic testing for RV, AV, and NV is needed in the primary care setting for the children management.

In Italy, the pediatric primary health care level is usually the family pediatrician (FP). We used the* Pedianet* network of family pediatricians (http://www.pedianet.it) to collect data and understand the disease burden of community-acquired RGVE, AVGE, and NVGE. The burden of AGE disease was also analyzed in terms of social impact for the families estimating the indirect cost caused by workdays lost.

*Aims*. The primary aim of this study was

- to estimate the incidence of RVGE leading to a FP visit among children < 60 months (5 years) of age in a well-defined Italian population.

The secondary aim of this study was

- to estimate the incidence of NVGE and AVGE leading to a FP visit among children < 60 months of age in a well-defined Italian population;
- to determine the age of the children, seasonal distribution, and disease severity of RVGE, NVGE, and AVGE among children < 60 months;
- to compare the outcomes in RV, NV, and AV positive and negative children with AGE;
- to estimate the medical and societal burdens of RVGE, AVGE, and NVGE on FPs practice and families.

## 2. Material and Methods

This observational, prospective, FP-based study used an established Italian network (*Pedianet*) covering a well-defined number of patients (i.e., those registered by each individual FP) in the Veneto Region of Italy.

Surveillance was conducted for 12 months from May 2010 to April 2011 (including a full RV season, 2010-2011) to assess disease incidence rates, disease age distribution, disease severity, seasonal variations in disease burden, and costs of viral AGE.

### 2.1. Study Setting

The city of Padua and nearby residential town where about 16000 children < 60 months (5 years) are living has been chosen. A number of FPs following between 7000 and 10000 children < 60 months of age were involved in the study.

All children < 5 years of age from the defined population who presented at the selected FP sites with AGE (as defined below) were included in the study.

A child was considered eligible for the study if she/he met the following criteria:

- A male or female child < 60 months (5 years) of age at the time of the FP visit.
- Child belonging to the population under surveillance selected for the study (served by the FP sites selected for the study).
- Child brought to FP for AGE during the study period.
- Written informed consent by parent/guardian of subject.

The following were considered exclusion criteria:

- Children aged > 60 months (5 years).
- Previously diagnosed chronic gastrointestinal tract disease where symptoms were similar to those of AGE.
- Known nosocomially acquired RVGE.
- No written informed consent.
- Children not living permanently in the study area.

Their parents/guardians were asked to consent to participate and to have a stool sample collected and tested.

An RVGE, AVGE, and NVGE case was defined as an AGE case (corresponding to the AGE case clinical definition) with RV, AV, or NV positive detection by PCR.

The database used for the analysis contains all and only the AGE cases enrolled for this study.

Incidence of AGE cases has been described as both number of cases and rate. Such figures will be stratified by age group at onset and calendar month of onset.

The database used for the analysis included data about patients' age, gender, RV vaccination, and AGE signs and symptoms (such as fever > 38°C, vomit, abdominal pain, convulsion, lethargy, and dehydration).

For every episode also the following were collected:

- Length of the episode.
- In case the AGE led to hospitalisation: the length of stay in hospital and hospitalisation diagnosis (ICD-9).
- If AGE led to emergency room (ER): the number of ER access times.
- In case of similar disease among family members: if father/mother had to take days off work and the number of days of work lost.

Data included in the* Pedianet *database followed all the Italian privacy regulations and laws.

Patients providing data to* Pedianet *and stool samples for the present study had to give informed consent before their anonymous information could be included in the dataset.

### 2.2. Statistical Analysis

Incidence rates of RVGE, AVGE, and NVGE were calculated as the ratio between cases and person-time.

Person-time was calculated as the average of the total population aged less than 60 months (5 years), registered with the FPs enrolled for the study, at the beginning and the end of the surveillance period. Poisson exact 95% confidence intervals will be calculated.

RVGE, AVGE, and NVGE incidence rates were stratified by age of the children and month of onset. Age-specific incidence rates were calculated as the ratio between the number of cases that occurred in the age group and the person-time contributed by the children of the age group. Month-specific incidence rates were calculated as the ratio between the number of cases that occurred in that given month and the total person-time.

Rates of hospitalisations were compared by means of logistic regression, adjusted by age. Hospitalisation will be the dependent variable and RVGE (or AVGE or NVGE, resp.) the “exposure.” The role (as confounders) of the characteristics reported at baseline will be investigated entering such variables in the models. The logistic regression will produce an estimate of the odds ratio of the risk of hospitalisation.

The same approach was used to compare the access to the ER as well as the combined event “hospitalisation or ER.”

Comparison of length of stay in hospital was carried out by means of *t*-tests.

In order to estimate societal costs due to RV, AV, and NV, new variables were built which combine parents' absence from work (WORK_ABS and DAYS_ABS). WORK_ABS were used in the logistic models described in the previous paragraph whereas DAYS_ABS were compared through *t*-tests.

The cases for which the stool sample was not taken (or not processed by the lab) were excluded from the analysis. However, the number of such cases will be reported.

## 3. Results

### 3.1. Description of Study Population

Twelve FPs were included in the analysis accounting for 7239 person-years.

**Figure 1:**
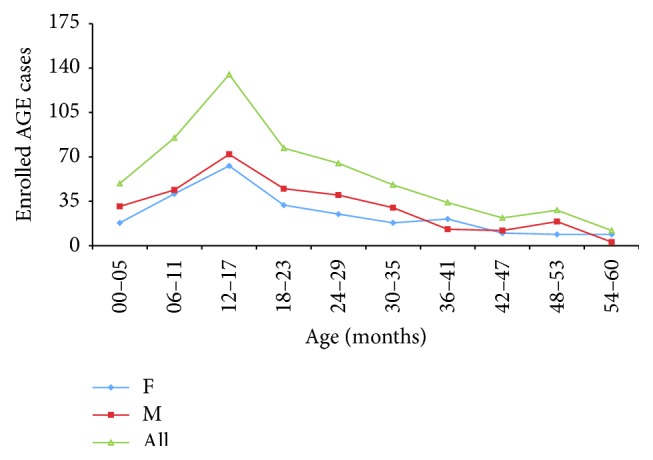
Age distribution of the cohort of children with AGE.

Five hundred fifty-five children < 60 months (5 years) of age presented at the selected FP sites with AGE, with 55,6% (*n* = 309) being male. The age distribution showed a peak between 6 and 18 months of age. Children < 24 months of age accounted for 62% of cases (Figure 1).

Two hundred fifty-two (45%), 269 (48%), and 362 (65%) cases over 555 children with gastroenteritis reported fever > 38°C, vomiting, and abdominal pain at enrolment, respectively. Only 3 cases (0.005%) reported convulsion and 17 cases (0.03%) presented with lethargy. The majority of children (82%) presented with no dehydration and 16% with mild (0–5%) and only 2% with moderate (5–10%) dehydration.

Only 3 children with gastroenteritis (0.5%) were vaccinated against rotavirus.

Collection of stool sample was performed in 460 (83%) of gastroenteritis cases.

### 3.2. Primary Endpoint

#### 3.2.1. Incidence of RVGE

Seventy-five cases resulted in being positive to rotavirus. None of them was vaccinated against RV. Estimated incidence rate was 1.04 per 100 person-years (95% CI: 0.81–1.30).

### 3.3. Secondary Endpoint

#### 3.3.1. Incidence of Adenovirus and Norovirus

One hundred twenty-six cases resulted in being positive to AV (two of them were vaccinated against RV) with an estimated incidence rate of 1.74 per 100 person-years (95% CI: 1.45–2.07) while 109 cases resulted in being positive to NV (one of them was vaccinated against RV) with an incidence rate of 1.51 per 100 person-years (95% CI: 1.24–1.82).

#### 3.3.2. Age of Children, Seasonal Distribution, and Diseases Severity of RVGE, AVGE, and NVGE

*RVGE* incidence was higher in the first 23 months of life with the higher incidence (3.60) in the range from 12 to 17 months.* AVGE* and* NVGE* mostly occurred in children < 41 and 29 months of life, respectively, with the same peak from 12 to 17 months of age.

**Figure 2:**
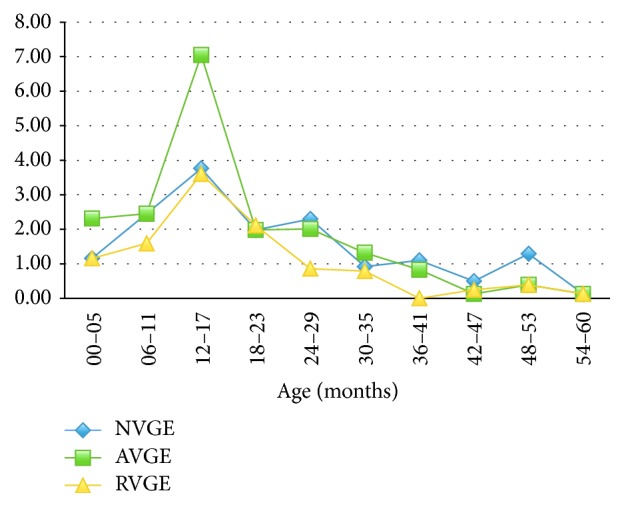
Incidence of AGE by age at onset.

Incidence by month of onset showed higher values for RVGE in November and from January to April, for AVGE from October to December, and for NVGE from October to June (Figure 2).

**Figure 3:**
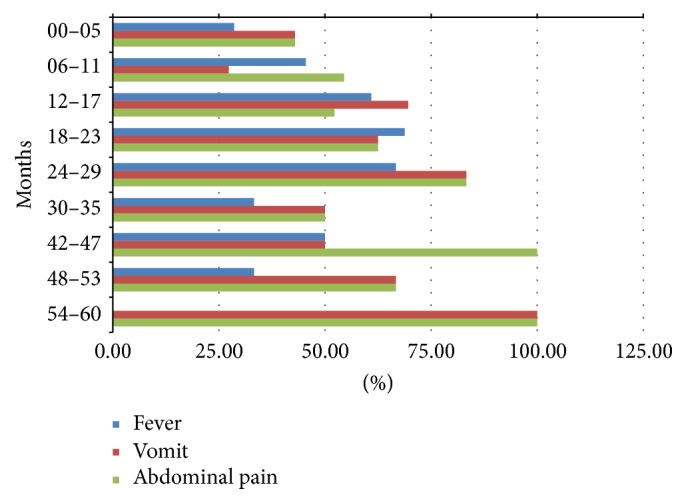
Distribution of fever, vomit, and abdominal pain during RVGE episodes stratified by age.

Association with fever > 38°C, vomit, and abdominal pain was present in 58%, 59%, and about 59% of cases, respectively. Distribution of symptoms during RVGE episodes is shown in (Figure 3). Convulsion occurred only in 1.3% of cases at enrolment, exclusively in young children from 6 to 11 months of age. Other neurological signs as lethargy occurred in 4% of cases, affecting children aged 6–17 months.

In AVGE group, association with fever > 38°C was present in about 44% of cases, vomiting in about 51% of cases, and abdominal pain in 61%. Neurological signs as convulsion and lethargy occurred in 0.8 and 1.6% of AVGE, respectively.

Fever occurred in 42% of cases, vomiting in about 62% of cases, and abdominal pain in 68% of patients affected by NVGE. Neurological signs as convulsion and lethargy occurred in 0.9 and 2.8% of NVGE, respectively.

**Figure 4:**
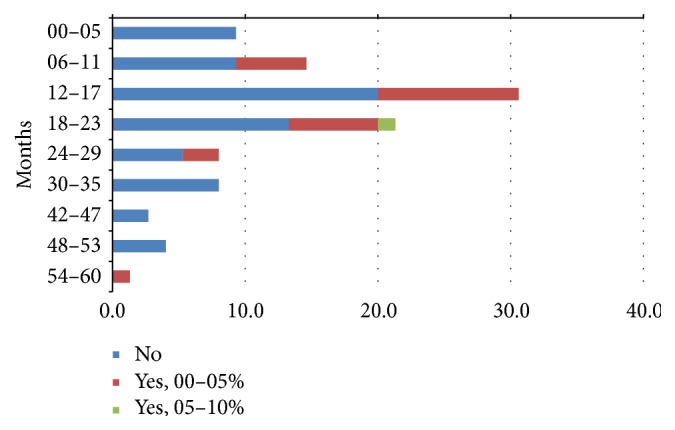
Distribution of dehydration during RVGE episodes stratified by age.

Fifty-four of RVGE cases (72%) were with no dehydration. Mild dehydration was reported only in about 27% of children. Children from 0 to 5 months did not experience dehydration. Only 1 child aged 18–23 months (1%) over the total of cases had moderate dehydration (Figure 4).

Mild dehydration occurred in about 18.3% of AVGE cases and 20% of NVGE cases. Moderate dehydration at enrolment occurred in 1.6% of AVGE and 0.9% of NVGE.

All these symptom and signs occurred independently of the age of children.

#### 3.3.3. Comparison between Outcomes in RV, AV, and NV Positive and Negative Children with AGE

None of 3 children vaccinated against rotavirus developed RVGE.

Mean length of all AGE episodes was 5.21 days (95% CI 4.94–5.47): 5.42 days (95% CI 5.53–4.78) for RVGE cases, 5.41 days for AVGE episodes, and 5.70 days for NVGE (Table 1).

**Figure 5:**
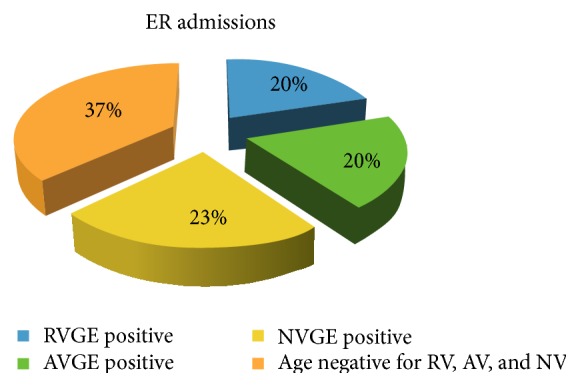
Comparison between ER access times during RVGE, AVGE, and NVGE episodes.

Thirty-five cases of AGE needed admission to emergency room (ER); 7 of these cases were RVGE (20.0%). The mean number of access times to ER, restricted to all AGE cases that accessed the ER, was 1.09 with a mean of 1.14 access times for RVGE episodes. The proportion of AVGE cases that needed access to ER was 7 over 35 (20.0%) total cases with a mean number of access times to ER, restricted to cases that accessed the ER, of 1.14. The proportion of NVGE cases that needed access to ER was 8 over 35 (22.8%) total cases with a mean number of access times to ER, restricted to cases that accessed the ER, of 1.13 (Figure 5).

**Figure 6:**
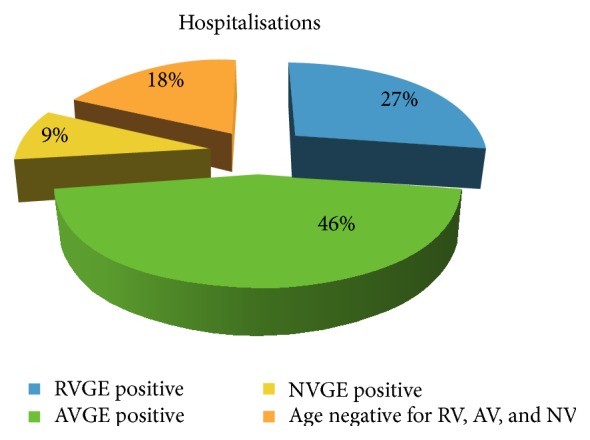
Comparison between hospitalisations during RVGE, AVGE, and NVGE episodes.

Hospitalisation occurred in 12 cases (2.54%) of all AGE cases and only 3 cases (25.0%) were RV positive (aged 6–17 months). Of 12 hospitalised children, 5 were AVGE (41.7%) and only 1 was a NVGE (8.3%) (Figure 6).

Mean length of hospital stay for all AGE cases was 1.08 days with shorter hospitalisation length for RVGE cases (0.67 days) whereas for AVGE it was 1.80 days and for NVGE, restricted to cases hospitalised, it was zero days.

All AGE cases hospitalised or that needed access to ER were 40 (17.5%) cases of RVGE, 9 (22.5%) cases of AVGE, and 8 (20.0%) cases of NVGE.

#### 3.3.4. RVGE

The comparisons between children who tested RV positive and those who tested RV negative showed an Age-Adjusted Mantel-Haenszel Relative Risk of hospitalisation of 1.857 (95% CI: 0.494–6.981) and a RR of ER access of 1.299 (95% CI: 0.588–2.871) with logistic regression RR of 1.145 (95% CI: 0.453–2.891). The Age-Adjusted Mantel-Haenszel RR comparing the rates of the combined event “hospitalisation or ER access” was 1.100 (95% CI: 0.505–2.397) and the logistic regression RR was 1.003 (95% CI: 0.406–2.473).

*t*-test comparing the hospitalisation lengths showed no statistically significant difference in RVGE admissions.

#### 3.3.5. AVGE and NVGE

The comparisons between children who tested AV positive and those who tested AV negative and children who tested NV positive and those who tested NV negative showed an Age-Adjusted Mantel-Haenszel Relative Risk of hospitalisation of 1.623 (95% CI: 0.560–4.698) and a RR of ER access of 0.640 (95% CI: 0.290–1.412) with logistic regression RR of 0.629 (95% CI: 0.257–1.536) for AVGE and an Age-Adjusted Mantel-Haenszel Relative Risk of hospitalisation of RR = 0.301 (95% CI: 0.039–2.307) and a RR of ER access of 1.005 (95% CI: 0.477–2.116) with logistic regression RR of 1.055 (95% CI: 0.451–2.464) for NVGE.

The Age-Adjusted Mantel-Haenszel RR comparing the rates of the combined event “hospitalisation or ER access” for AVGE was 0.727 (95% CI: 0.360–1.471) and the logistic regression RR was 0.729 (95% CI: 0.328–1.620) and for NVGE the Age-Adjusted Mantel-Haenszel RR was 0.845 (95% CI: 0.407–1.758) and the logistic regression RR was 0.814 (95% CI: 0.357–1.858).

*t*-test comparing the hospitalisation length for AVGEs showed statistically significant difference between AVGE and not AVGE admissions while for NVGE it was not possible to be performed.

#### 3.3.6. Medical and Societal Burdens due to RVGE, AVGE, and NVGE on FPs and Families

Proportion of cases with family members affected by AGE and the mean working days lost by the parents and by the mother, restricted to the cases in which at least one parent had to take days off, are summarized in Table 2.

**Figure 7:**
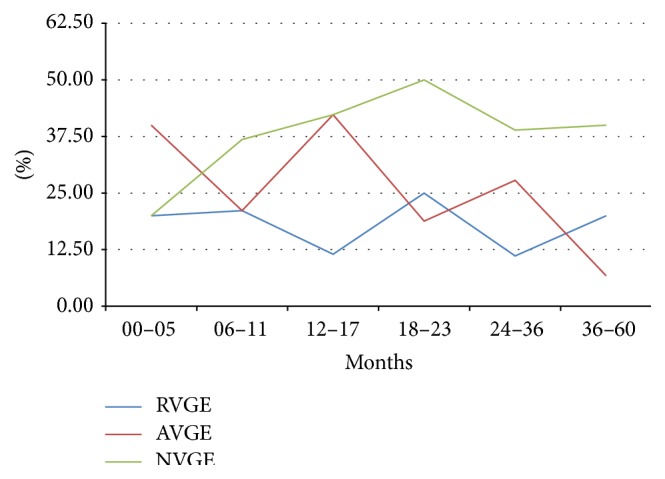
Proportion of cases with family members affected during RVGE, AVGE, and NVGE episodes stratified by age.

For RVGE cases, the percentage seems to be higher in younger (0–11 months) and older (36–60 months) affected children (Figure 7).

Proportion of family members affected was higher in NVGE cases than in not NVGE cases (22.03).

For RVGE, Age-Adjusted Mantel-Haenszel RR comparing the rates of absence from work was 0.976 (95% CI: 0.565–1.689) and the logistic regression RR was 0.860 (95% CI: 0.435–1.702). *t*-test comparing the working days lost showed no statistically significant difference.

Also for AVGE and NVGE, Age-Adjusted Mantel-Haenszel RR comparing the rates of absence from work was 1.125 (95% CI: 0.721–1.755) for AVGE and 1.460 (95% CI: 0.944–2.260) for NVGE with logistic regression RR of 1.172 (95% CI: 0.676–2.033) and 1.570 (95% CI: 0.908–2.716), respectively. In both cases, *t*-test comparing the working days lost showed no statistically significant difference.

## 4. Discussion

Twelve family pediatricians (FPs) were involved from May 2010 to May 2011 in the Pedianet network.

In the period of study, 555 children < 60 months of age presented at the selected FP sites with AGE. The age distribution was skewed as expected with a peak between 6 and 18 months of age. Children < 24 months of age accounted for 62% of cases.

Seventy-five cases resulted in being positive to RV with an estimated incidence rate of 1.04 per 100 person-years, lower than AVGE and NVGE rate in Italian children < 5 years old with AGE belonging to the Pedianet database. In the EU, the annual incidence of community-acquired RVGE among children < 5 years of age has been reported ranging from 1.33 to 4.96 cases per 100 person-years [36–39] and even 12-fold higher among children under three years of age [40]. The REVEAL study reported that RVGE in children < 5 years of age is responsible for between 53.0% and 68.9% of cases presenting to hospitals, 35.4% and 63.3% of those seen in emergency departments, and 7.7% and 41.3% of cases seeking primary care physicians [41]. A second prospective European multicentric study reported that the overall proportion of community-acquired rotavirus infections was 43.4%, with most of these cases (80.9%) occurring in children under 2 years of age [42]. RVGE incidence obtained in our study is comparable to the lower value reported from previous European studies and probably could be underestimated as many patients received care at home without being referred to a FP. Indeed, a high number of children with RVGE are not sick enough to be admitted to the hospital and many patients receive no medical care at all [11]. For Europe, estimations of the proportion of RVGE patients receiving no medical care ranged from 25% to 51% of patients [38, 43, 44]. Two studies from a day care setting in France reported that 34.6% and 14.3% of RVGE cases, respectively, did not seek medical attention [40].

RVGE incidence was higher in the first 23 months of life with the highest incidence in the range from 12 to 17 months shortly before being reported [41–44]. NVGE and AVGE showed the same trend confirming the high AGE's burden in children younger than two years.

As it is well known, the RV infection peak occurs in the winter season between November and February in temperate climates [5, 15, 16]. The low incidence in December for all the viral agents could be explained considering lower rate of children attending FP. In fact, in this time of the year, FP consulting rooms are closed and there is an increase in ER visits and subsequent hospital admissions.

Signs and symptoms of AGE are similar when stratified by age and independently from the viral pathogen. Still debated is the proportion of children with dehydration due to acute RVGE.

In our report, 72% of RVGE cases presented with no dehydration. The REVEAL study reported that the proportion of children with dehydration due to acute RVGE varied between 11.1% and 71.4%, and in most countries it was considerably higher than for those with rotavirus-negative disease [41]. The results of our study could be comparable with the REVEAL lower value, but it is even less considering what has been reported by Forster et al. where dehydration is evident in 75.7% of patients with RVGE and is severe in 11.3% of them [42]. It is likely that this rate variation depends on whether patients are seen in hospital, ER, or primary care physician clinic. Only 18.3% and 1.6% of children with AVGE presented mild and moderate dehydration, respectively, whereas NVGE was associated with mild dehydration in about 20% and moderate dehydration in 0.9% of cases. Compared with other viral causes studied, mild dehydration was reported in a slightly higher proportion of children with RVGE but moderate or severe dehydration was unlikely to occur, according to a previous Italian community-based study which showed that dehydration at initial presentation in primary care was associated with a higher likelihood of RVGE (OR: 1.8) [45]. Also in this case data presents large variability, because in some countries proportion of children with dehydration is comparable among children with or without RVGE [41].

In the analyzed period, the mean length of all AGE episodes was 5.21 days and 5.42 days for RVGE cases. Hospitalisation occurred in 2.54% of AGEs and only 3 cases were RV positive. All AGE cases hospitalised or those that presented to the ER were 40 and 7 of these were RVGEs. In EU, hospital stay due to acute RVGE ranges from 2.5 days to 5.0 days [41, 46]. In the REVEAL study, the proportions of hospital and emergency referrals among children presenting at primary care with acute RVGE ranged from 13.0% to 57.1% and from 6.1% to 45.3%, respectively, for all countries included in the study [41]. Additional country-specific studies show different hospital admission rates for community-acquired disease due to acute RVGE (France 81% [47]; Germany 7% [40]; Italy 11.2% [48]). In Greece, hospital admissions due to RVGE are significantly more frequent than non-RVGE (51.4% versus 22% nonrotavirus; *P* < 0.01) [49].

Overall reported hospitalisation due to RVGEs is longer than that for other viral gastroenteritis cases [39, 50] but our *t*-test data analysis comparing the hospitalisation lengths showed no statistically significant difference in RVGE admissions.

In addition, hospitalisation or/and access to the ER were not significantly different in RVGE if compared with NVGE and AVGE but, according to literature, comparing AVGE and non-AVGE admissions the hospitalisation lengths showed statistically significant difference [42, 43]. Indeed, children with AVGE are more likely to present diarrhoea that usually lasts more than in RVGEs and consequently are more likely to require hospitalisation [31, 32].

The RVGE heavy economic burden including direct costs (e.g., medical consultation or hospitalisation) and elevated indirect costs (e.g., parent workdays lost) [12, 15, 16]. Proportion of cases with family members affected by AGE was 22.03%. For RVGE cases, the percentage rose to 24% while, in the NVGE group, 37.6% of patients had a family member affected. In this study, there was no significant difference between RVGE, AVGE, and NVGE in terms of parents' workdays lost. This is consistent with what was previously reported in the literature that NVGEs usually have a high attack rate, but symptoms are milder and last less than in other viral AGEs [20, 21].

Features of RVGE in terms of hospitalisation length and indirect cost are lower than reported in previous studies [12, 15–17].

Noteworthy is the RV vaccination coverage rate in our cohort: only 3 out of 555 children had received a universal RV vaccine 5 years after its release.

Actually, only 5 regions have officially recommended RV vaccination, with a wide range of offered solutions [50]. In 2 regions (Lazio and Tuscany), vaccination is offered to all infants with a copayment system; in Basilicata and Piedmont, it is free for preterm infants and high-risk groups and in Apulia children can receive RV vaccination for free based on a FP's request. In the Veneto Region it is strongly suggested but not included in the vaccinations plan, so families have to pay to receive it [50].

Analyzing the possible reasons of low RV vaccination diffusion, the most common barriers are represented by the low perception of RV disease burden, potential safety concerns, and unfavorable cost-effectiveness. A primary care network for AGE surveillance could be a useful tool to increase RV disease awareness and to follow the rate of infection after RV vaccination implementation. Indeed, the main strength of this study is that data have been collected from Pedianet, a reliable network of FPs working within the Italian National Health Service. This allowed calculating the incidence of AGE, RVGE, AVGE, and NVGE on the basis of person-time by age groups and provides a precise picture of the disease in that period of time. Furthermore, in this prospective study, which had strict inclusion and exclusion criteria, the aetiology of AGE has been confirmed by PCR stool testing for almost all of the patients.

One limitation of the study is that PCR analyses on stool were performed only to detect presence or absence of different viruses but RV different genotypes have not been analyzed. This specific exam could be a very efficient tool for monitoring the RVGE aetiology especially in those regions where the vaccination is offered.

The duration of the study represents another limitation, considering that a one-year study period is too short to exclude interannual and seasonal variability with consequent risk of over- or underestimating the burden of infection.

Finally, in this study, AGE episodes not seen by FPs but diagnosed in an ER have not been recorded. However, as FPs are free of charge, it is likely that only a negligible proportion of children acceded directly to ER. The only exception is represented by the holidays when FP consulting rooms are closed and consequently the ER visits increase.

## 5. Conclusion

Results of the present study reflect the large variability of data present in the literature regarding the public health and economic impact of RVGEs in the EU, comparing them also with AVGE and NVGE, especially in the postvaccination era. This observation underlines the usefulness of primary care networks for AGE surveillance and further studies of community-acquired gastroenteritis in children.

## Figures and Tables

**Table 1:** Mean length of RVGE, AVGE, and NVGE episodes.

	RVGE						AVGE						NVGE							All	
		No			Yes			No			Yes			No			Yes				
	Mean	95% CI		Mean	95% CI		Mean	95% CI		Mean	95% CI		Mean	95% CI		Mean	95% CI		Mean		95% CI
00–05 months	5.37	4.33	6.41	4.57	1.91	7.23	5.29	3.98	6.59	5.14	3.93	6.36	5.11	4.06	6.17	5.86	3.5	8.21	5.24	4.31	6.17
06–11 months	5.4	4.64	6.15	6.45	3.09	9.82	5.4	4.54	6.27	6.06	4.2	7.92	5.23	4.36	6.09	6.65	4.86	8.44	5.55	4.78	6.33
12–17 months	5.59	4.91	6.28	5.65	4.33	6.97	5.48	4.71	6.25	5.84	4.81	6.88	5.55	4.89	6.22	5.79	4.3	7.28	5.6	5	6.2
18–23 months	4.69	4.17	5.2	5.5	3.79	7.21	4.83	4.17	5.48	5.07	4.05	6.08	4.56	4.08	5.04	6	4.16	7.84	4.88	4.33	5.43
24–36 months	4.56	4.06	5.07	5.17	3.2	7.14	4.55	3.99	5.1	4.92	3.78	6.05	4.38	3.89	4.86	5.48	4.07	6.89	4.64	4.15	5.13
36–60 months	5.24	4.56	5.92	5.33	3.75	6.91	5.37	4.64	6.09	4.55	3.58	5.51	5.42	4.59	6.24	4.87	3.94	5.8	5.25	4.62	5.88
All	5.14	4.87	5.42	5.53	4.78	6.29	5.13	4.83	5.44	5.41	4.9	5.92	5.06	4.77	5.35	5.7	5.11	6.29	5.21	4.94	5.47

**Table 2:** Societal burdens due to RVGE, AVGE, and NVGE.

	All AGE	RVGE	AVGE	NVGE
Family members affected (%)	22.03	24	22.22	37.61
Mean working days lost by parents	0.56	0.60	0.74	0.71
Mean working days lost by mother	3.30	3.46	3.88	3.21

## Abbreviations

AGE: Acute gastroenteritis
AV: Adenovirus
AVGE: Adenovirus AGE
DMP: Data Management Plan
ER: Emergency room
EU: European Union
NV: Norovirus
NVGE: Norovirus AGE
RV: Rotavirus
RVGE: Rotavirus gastroenteritis
SAP: Statistical Analysis Plan
SD: Standard deviation
95% CI: 95% confidence interval.
